# MedTAG: a portable and customizable annotation tool for biomedical documents

**DOI:** 10.1186/s12911-021-01706-4

**Published:** 2021-12-18

**Authors:** Fabio Giachelle, Ornella Irrera, Gianmaria Silvello

**Affiliations:** Department of Information Engineering, University of Padua, Padua, Italy

**Keywords:** Biomedical annotation tools, Entity extraction, eHealth, Digital health

## Abstract

**Background:**

Semantic annotators and *Natural Language Processing (NLP)* methods for *Named Entity Recognition and Linking (NER+L)* require plenty of training and test data, especially in the biomedical domain. Despite the abundance of unstructured biomedical data, the lack of richly annotated biomedical datasets poses hindrances to the further development of NER+L algorithms for any effective secondary use. In addition, manual annotation of biomedical documents performed by physicians and experts is a costly and time-consuming task. To support, organize and speed up the annotation process, we introduce MedTAG, a collaborative biomedical annotation tool that is open-source, platform-independent, and free to use/distribute.

**Results:**

We present the main features of MedTAG and how it has been employed in the histopathology domain by physicians and experts to annotate more than seven thousand clinical reports manually. We compare MedTAG with a set of well-established biomedical annotation tools, including BioQRator, ezTag, MyMiner, and tagtog, comparing their pros and cons with those of MedTag. We highlight that MedTAG is one of the very few open-source tools provided with an open license and a straightforward installation procedure supporting cross-platform use.

**Conclusions:**

MedTAG has been designed according to five requirements (i.e. available, distributable, installable, workable and schematic) defined in a recent extensive review of manual annotation tools. Moreover, MedTAG satisfies 20 over 22 criteria specified in the same study.

## Background

In the last decades, exascale volumes of biomedical data have been produced, where the vast majority is available as unstructured text [1]. Health-care professionals traditionally rely on free-text reporting for communicating patient information such as diagnosis and treatments. For instance, narrative clinical reports are usually conceived as free-text reports, which are human-readable but not machine-readable. This brings interoperability issues and limitations to effective secondary reuse of data, essential for medical decision making and support. In order to process the vast amount of unstructured biomedical data from clinical reports and *Electronic Health Records (EHRs)*, *Information Extraction (IE)* algorithms and NLP techniques have been developed and are currently exploited.

To this aim, significant efforts have been dedicated to applying *Named Entity Recognition and Linking* (NER+L) methods for entity extraction and semantic annotation [2–6]. Semantic annotation is the NLP task of identifying the type of an entity and uniquely linking it to a corresponding knowledge base entry [7]; it leverages both text-processing and *Machine Learning (ML)* techniques to tackle biomedical information extraction challenges such as terms and abbreviations disambiguation. Furthermore, semantic annotation tasks based on ML methods are often carried out in a supervised context where large-scale training and test annotated corpora are required. Moreover, even in an unsupervised context, NER+L models require annotated datasets for evaluation purposes. However, the lack of manually annotated biomedical datasets poses hindrances to the further development of NER+L systems. In addition, most of the training data available for the biomedical domain covers mainly common entity types (e.g., drugs, genes, and diseases) [8–11], thus the coverage of some biomedical sub-domains is limited. For these reasons, several attempts to create large annotated biomedical corpora have been conducted [12–19].

To achieve the high-quality standards required for the biomedical domain, the annotation process demands human-expert supervision. Nevertheless, manual annotation of large datasets is an expensive and time-consuming task requiring plenty of expert annotators with extensive experience in biomedical contents. To support, organize and speed up the annotation process, several annotation tools have been developed [20–33]. However the biomedical domain is particularly challenging, since biomedical texts contain mentions that are burdensome for semantic annotation, such as the abbreviations of genes and proteins. Moreover, the specificity of some biomedical sub-domains, such as histopathology, requires fine-grained annotation systems designed to be customizable according to physicians’ and experts’ needs.

**Fig. 1:**
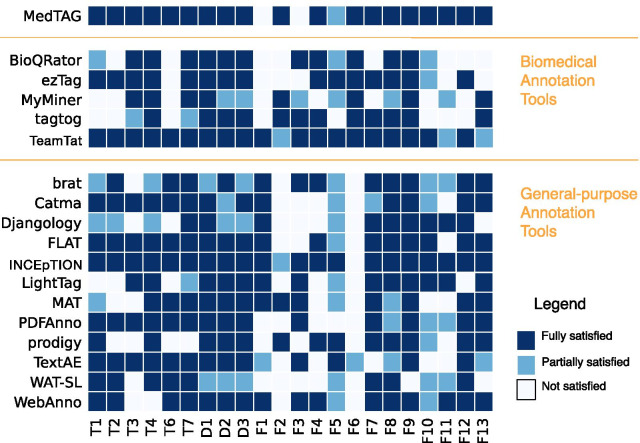
Overview of annotation tools and their functionalities. The annotation tools considered come from a recent extensive review of tools for manual annotation of documents [34]. In addition, we consider also TeamTat [35] and INCEpTION [36] and report our judgements. The annotation tools are assessed with 22 criteria, defined in the latter review study, among three categories: *Data* (D), *Functional* (F) and *Technical* (T). The fulfillment of each criterion is indicated with a color in a three levels scale: white (feature absent or not met), light blue (feature partially satisfied), blue (feature satisfied)

In recent years, several biomedical annotation tools have been released [34, 37]. Motivation for the wide variety of biomedical annotation tools available could be the necessity of domain-specific functionalities that might be only partially supported or not by other well-established tools. Hence, some tools could be handier than others for a specific task of interest.

A recent extensive review of both general-purpose and biomedical annotation tools provides a detailed comparison of state-of-the-art annotation tools [34]. Some of the common limitations of the available tools are, for instance, the non-availability of the source code or the raised exceptions and failures during the installation process. In addition, even the most popular annotation tools present drawbacks such as a burdensome installation procedure or the lack of documentation. As an example, WebAnno [38] and brat [39] are popular general-purpose annotation tools with a comprehensive set of functionalities, but their installation process is rather complex for the not technology-savvy users. INCEpTION [36, 40] is a more recent general-purpose annotation tool from the authors of WebAnno [38], that mitigates this issue with a web service enabling the users to work online. Moreover, general-purpose annotation tools often do not fulfill the needs of biomedical experts; thus, domain-specific solutions are preferable for this field. Even though brat [39] has been used in several biomedical projects [41–45], it is designed for general-purpose annotation, thus it provides additional features that are not suited for physicians and experts of the biomedical domain. Since the annotation process is a time-consuming task, biomedical annotation tools should be designed to offer an intuitive streamlined interface that minimizes redundant features, fulfill domain-specific requirements and reduce the annotators workload.

For the in-depth analysis, we focus on the tools specifically designed for biomedical annotations: BioQRator [25], ezTag [26], TeamTat [35], MyMiner [27] and tagtog [28]. Additionally, we also consider two general-purpose annotation tools that are used by the biomedical community as well - i.e., brat [39] and INCEpTION [36].

In Fig. 1, we can see a heat-map reporting on the functionalities of the current text annotation tools as analyzed by a very recent extensive survey [34]. The provided heat-map is to be used as a visual summary of the features provided by each annotation tool.^1^ In particular, the heat-map considers a list of 15 annotation tools selected according to five major requirements: (i) **Available**: the executable and project source code should be available; (ii) **Web-based**: the tool should be provided as an online web application or as an installable application running in a web browser container; (iii) **Installable**: the installation process should last two hours at most; (iv) **Workable**: it should work for hands-on experiments; (v) **Schematic**: users should be able to configure the annotation schema at will. Hence, several biomedical annotation tools such as Argo [29], Egas [24], Marky [30], ODIN [31], Pubtator [32] and Textpresso [33] are not considered since they do not satisfy one or more of the previous five requirements.

Moreover, the selected annotation tools are compared according to a set of 22 criteria chosen among the original 26 criteria of the same study [34]. In particular, the criteria are grouped in four categories: (i) *Data*, (ii) *Functional*, (iii) *Publication* and (iv) *Technical*. We excluded the publication criteria (i.e., the four missing criteria) since we are interested in comparing the facilities and functionalities provided by the different tools and not on their coverage in scientific publications.

The *data* criteria are: (D1) format of the schema – whether it is configurable or uses standard formats (e.g. JSON, XML); (D2) input format for documents – whether the input documents are based on standard formats (e.g. JSON, XML) and (D3) output format for annotations – whether the annotations are based on standard formats (e.g. JSON, XML).

The *functionality* criteria are: (F1) support for overlapping mentions/annotations; (F2) support for document-level annotations – users can specify the labels that apply to the whole document (not just for a textual portion); (F3) support for relationship annotations; (F4) support for ontologies and terminologies (i.e. a procedure to import terminology resources is provided); (F5) Support for built-in predictions and active learning from pre-annotated documents; (F6) Integration with PubMed – users can annotate PubMed abstracts just providing a list of PubMed ids; (F7) Suitability for full texts (i.e., tool capable of displaying long text correctly, without compromising readability); (F8) Allowance for saving documents partially (i.e., holding annotations partially to later continue the annotation process); (F9) Ability to highlight parts of the text; (F10) Support for users and teams; (F11) Support for *Inter-Annotator Agreement (IAA)*; (F12) Data privacy (i.e., can be used for private data); (F13) multilingual support (i.e., annotating multilingual documents, that might contain special characters).

The *technical* criteria are (T1) Date of the last version – whether the last version (or commit) has been released within the past five years; (T2) availability of the source code – whether the source code is available in version control platforms; (T3) online availability for use; (T4) easiness of installation – i.e., available online (no installation required) or easy and fast to install (up to half-hour time); (T6) license allowing modification and redistribution; (T7) free of charge. We excluded T5 (quality of documentation) from the technical criteria since we are interested in objective and assessable criteria.

Figure 1 shows that several tools lack one of the following functionalities: (i) document-level annotation; (ii) ontology and terminology resources support; (iii) support for multi-label annotation; and (iv) support for collaborative annotations with users and teams. Moreover, seven over the seventeen selected tools are provided through a license that limits modifications and redistribution.

To mitigate this, we introduce MedTAG, a customizable, collaborative, web-based annotation tool provided as a docker container to enable cross-platform support and quick and easy installation. MedTag provides a step-by-step schema configuration, by which the project/team leader can specify in detail which document parts or document fields can be annotated. We designed MedTAG according to the five primary annotation tools’ requirements previously discussed. Besides, we determined the feature coverage provided by MedTAG concerning the former criteria. Figure 1 shows that MedTAG satisfies most of the criteria, having a feature coverage of 20 criteria over 22. The rest of the criteria currently not covered by MedTAG, such as the relationship annotations and active learning capabilities, are planned as future work.

## Implementation

MedTAG has been designed to be flexible and customizable, so that users can easily install it and configure the annotation schema at will. Hence, MedTAG is not limited to a specific (sub-)domain (e.g., histopathology), but it can be seamlessly used in different biomedical sub-domains. The key MedTAG functionalities are: (i) a web-based collaborative annotation platform with support for users and roles; (ii) a user-friendly interface with support for click-away mention annotation, mentions highlighting in different colors and automatic saving every time an action is performed; (iii) sorting of documents based on the lexicographic order or the “unannotated-first” policy; (iv) support for mobile devices; (v) download of annotations in several formats (i.e., BioC/JSON, BioC/XML, CSV, JSON); (vi) support for multi-label annotation; (vii) support for document-level annotations; (viii) multilingual support; (ix) support for ontologies/concepts to use for the annotation process; (x) support for IAA; (xi) integration with PubMed; (xii) support for automatic built-in predictions; (xiii) support for schema configuration, so that users can easily import data (i.e., documents, labels and concepts), as CSV files, and choose which document fields to annotate. In order to achieve automatic annotations and built-in predictions, we integrated the *Semantic Knowledge Extractor Tool (SKET)*^2^ in MedTAG. Note that the support for built-in predictions is currently limited to three cancer use-cases (i.e., cervix, colon, and lung cancer). Nevertheless, we plan to extend the support for automatic built-in predictions also for other use-cases. General-purpose automatic annotation methods are of limited efficacy for the biomedical domain; nevertheless, the integration of SKET paves the road for the integration of other third-party libraries users may want to employ.

To exploit the concept linking functionality, MedTAG requires the admin user to specify, during the configuration phase, the CSV file containing all the concepts used for annotating the clinical reports. During the first configuration, the admin user is not defined yet, thus the configuration is handled by the *Test* user in *Test mode*, as described in the *Installation and customization* section. Figure 5.2 shows the configuration interface that allows the users to specify the CSV file for the concepts. Moreover, the users can choose whether to use the concepts of the ExaMode ontology^3^ (necessary for the automatic annotation module using SKET) or a set of concepts from a different ontology. Then, the concepts provided in the CSV file populates the MedTAG database and are integrated in the drop-down menu available to the user to select the concepts. Every concept defined in the provided CSV is uniquely identified with a concept IRI. Thus, users could use concepts defined in different ontologies at the same time. Since the CSV file with the concepts for the annotation process is provided by the admin user, the coherence of the data (e.g., the same concept mapping to more than one IRI from different ontologies) should be checked and enforced by the admin herself. Nevertheless, in the case of the same entity mapping to different ontologies, MedTAG differentiates the concepts in the user interface based on the IRIs and other concept information such as use-cases and semantic areas. Thus, users have the means to disambiguate between potentially similar concepts.

MedTAG source code and the documentation are publicly available at this URL: https://github.com/MedTAG/medtag-core.

### Architecture

**Fig. 2:**
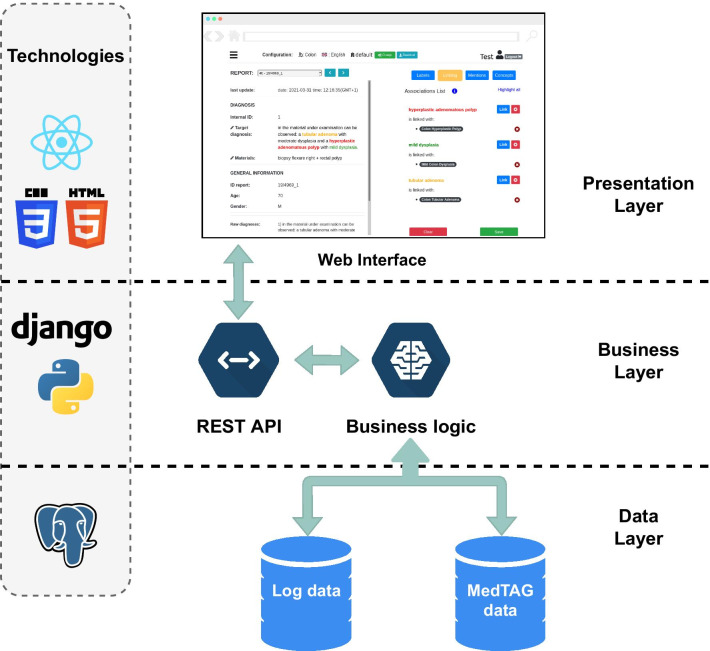
MedTAG Architecture. The data layer comprises two relational databases, namely, *MedTAG data* and *Log data* to store all the information concerning the annotation process (e.g., concepts, labels, reports, users and their annotations) and logging data such as notifications of malformatted clinical reports. The business layer comprises two business units: *Business logic* and *REST API* which jointly control the whole information flow from the front-end to the database and vice-versa. The presentation layer provides the MedTAG front-end, a web interface allowing users to annotate medical reports and download their ground truths

Figure 2 illustrates the MedTAG architecture, which consists of three logic layers (i.e., *Data*, *Business* and *Presentation* layer). The data layer concerns information and data management; it consists of two main relational databases realized with PostgreSQL, namely, the *MedTAG data* and the *Log data* databases. The former contains documents, entity concepts/labels, and the relations among them. The latter takes care of logging data such as user-provided information about issues with the documents to be annotated. The business layer controls the whole information flow as the information is displayed in the web interface and stored in the MedTAG database. It consists of two business units, the business logic, and the REST APIs end-point. The first one consists of Python routines and a controller that invokes the proper routine based on the received request. The second one is the back-end entry-point of MedTAG; it handles all the user requests from the web interface, invoking the business logic controller and returning its result to the front-end. The presentation layer provides the MedTAG front-end; it consists of a web interface to navigate the documents, annotate them and download the annotations in different formats (i.e., BioC/JSON, BioC/XML, CSV, and JSON).

Figure 2 shows the technologies adopted for each logic layer: (i) the front-end interface built with React.js,^4^ HTML5 and CSS3; (ii) the back-end for web API and services built with the Python web framework Django^5^; (iii) the *MedTAG data* relational database implemented using PostgreSQL.

Due to the multitude of architecture components, manually installing and configuring each one would be cumbersome and error-prone. To mitigate this, we provide a fast and reliable installation by distributing MedTAG as a docker container.

### Installation and customization

Since MedTAG is provided as a Docker container, both *docker*^6^ and *docker-compose*^7^ are required. The detailed installation procedure is described at https://github.com/MedTAG/medtag-core/tree/main#installation. We can summarize the MedTAG installation in three steps:

1. Check the Docker daemon - i.e., dockerd - is up and running.
2. Download the MedTAG_Dockerized^8^ folder from the medtag-core^9^ repository, or clone it.
3. Open the MedTAG_Dockerized project folder and, on a new terminal session, type docker-compose up.

Once the installation process has been completed, MedTAG is available on your browser at http://0.0.0.0:8000. At this stage, users can access MedTAG only in *Test mode* – i.e., by using the pre-loaded documents. The pre-loaded documents for the test mode are taken from the histopathology domain because we chose this domain as a use case for introducing and testing MedTAG functionalities.

Users can log into MedTAG and test it with the preloaded medical reports using *Test* as username and password.

**Fig. 3:**
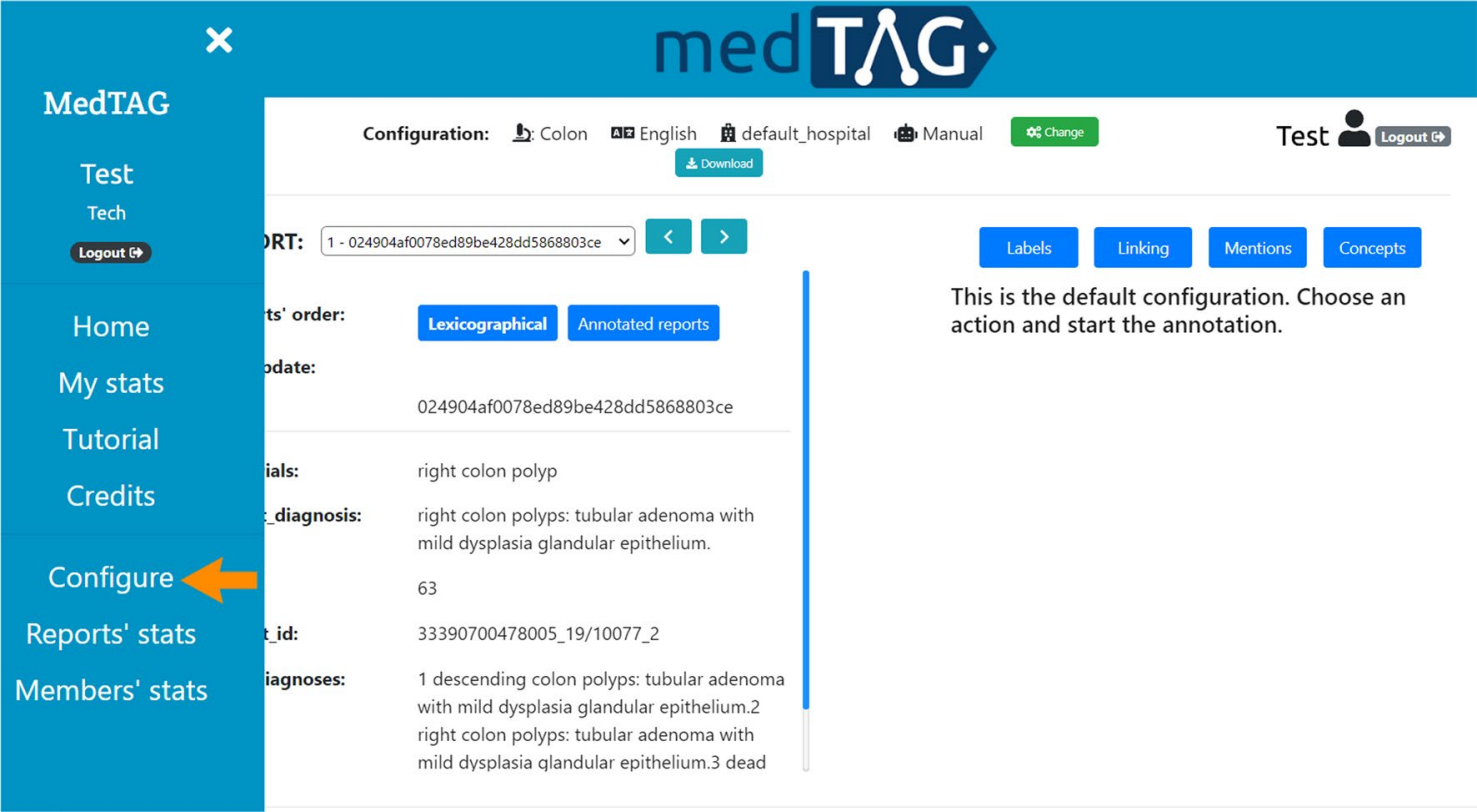
MedTAG sidebar provides the *Configure* option, indicated by the orange arrow, to set up a new custom configuration

**Fig. 4:**
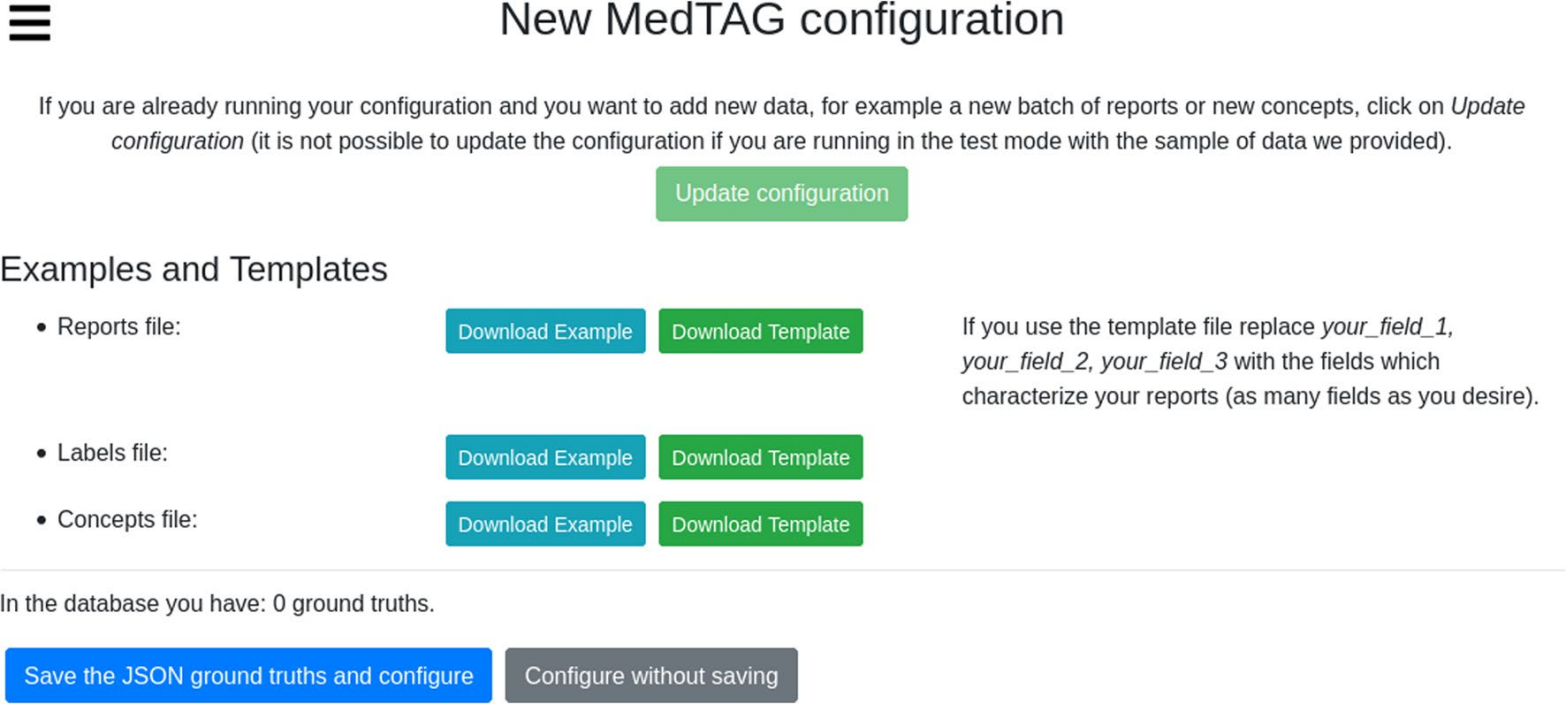
MedTAG new configuration interface allows the user to save the current data before creating a new configuration. To guide the user in providing the new configuration files needed (i.e. reports/documents, labels and concepts), MedTAG provides both example and template files. In particular, users can use the example files to test MedTAG without providing their own data. Instead, users can use the template files as a reference to structure their own configuration files

**Fig. 5:**
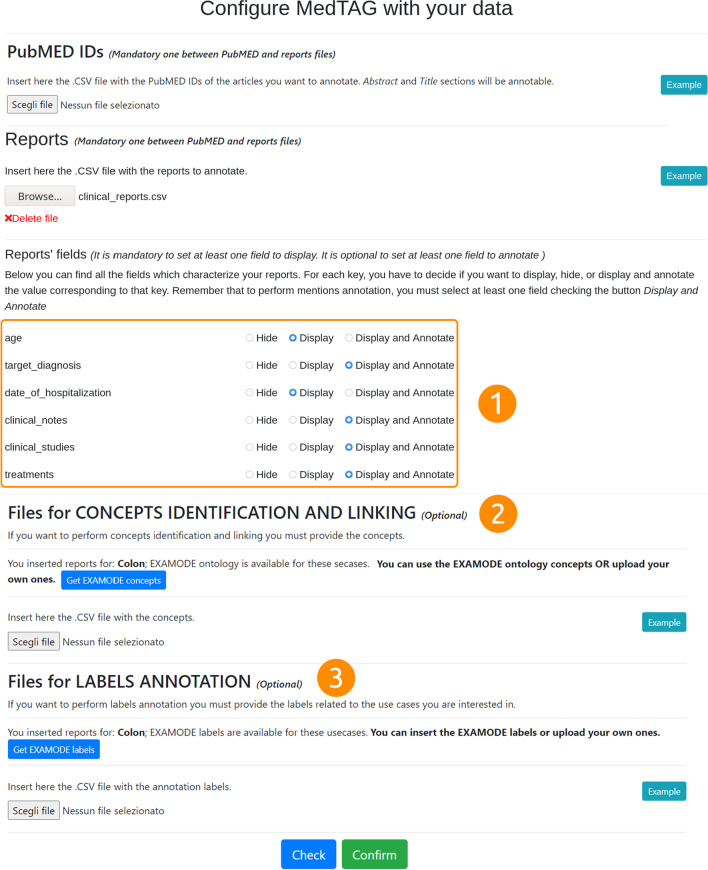
MedTAG main interface for data configuration. Users can provide their own CSV files for the reports/documents to annotate and the concepts and labels to use for the annotation process. Moreover, MedTAG detects automatically the document fields and allows users to specify which of them to annotate and/or display in the interface, as shown in the orange box (1)

To customize MedTAG, the users need to follow three steps: (i) open the menu and click on *Configure*, as shown in Fig. 3; (ii) follow the instructions of the guided procedure – i.e., users are asked to provide both the admin user credentials and three CSV files: concepts_file, labels_file and reports_file, as shown in Fig. 4. The users are provided with CSV templates and with examples containing real data to speed-up the data preparation procedure; (iii) choose which document fields to display and annotate as shown in Fig. 5; the *Check* button activates the file compliance procedures that will produce some state messages in different colors to inform the user about whether the CSV files provided are well formatted or not. Figure 5 shows the configuration interface that allows the users to specify whether to use the ExaMode concepts (indicated with number two) and labels (indicated with number three) or to upload a new set of concepts from different ontologies. The latter are necessary in case users want to take advantage of automatic annotation features. In addition, users can choose whether to annotate custom documents or PubMed abstracts and titles. In the first case, users are required to provide all the reports to annotate as a CSV file, that is, reports_file. Then, users can choose the report fields to annotate at will. In the second case, users have to specify a list of PubMed identifiers as a CSV file. Then, users can annotate both abstract and title of each PubMed article specified.

The detailed customization procedure is available at https://github.com/MedTAG/medtag-core#customize-medtag.

### User interface and interaction

**Fig. 6:**
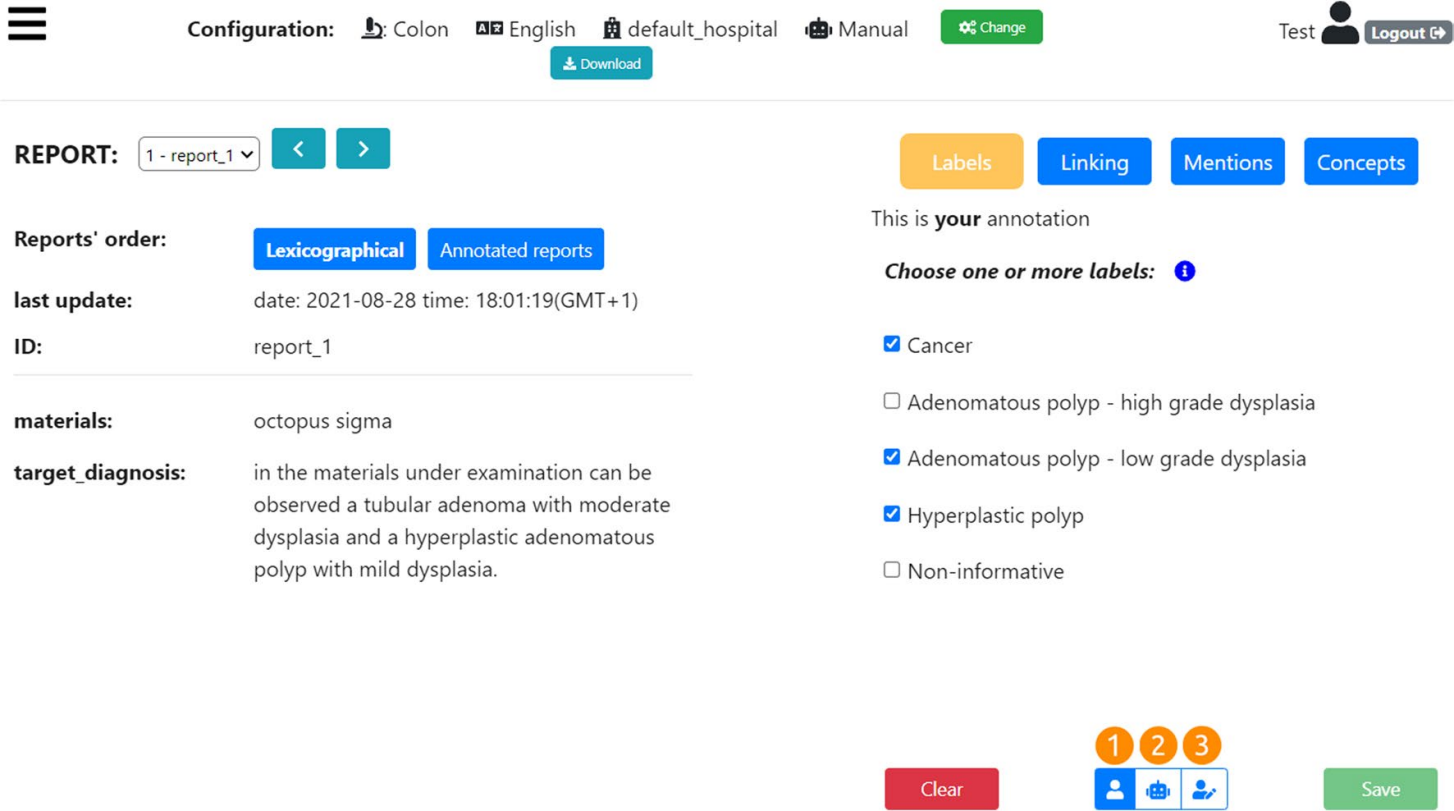
MedTAG main interface in test mode with default configuration: clinical case set to “Colon cancer”, reports’ language set to English, reports’ institute/hospital set to “default_hospital” (the real name has been anonymized) and the annotation mode set to manual. The annotation type active is the *Labels* one. Three labels have been checked: (i) *Cancer*; (ii) *Adenomatous polyp - low grade dysplasia* and (iii) *Hyperplastic polyp*

The MedTAG web interface has been developed based on the positive feedback received from physicians and experts in the digital pathology domain where an instance of MedTAG - i.e., ExaTAG - has been released. Figure 6 shows the main MedTag web-interface for the annotation of medical documents or reports. On the top of the web page, there is the header section with the current MedTAG configuration: (i) the clinical case (e.g., Colon cancer); (ii) the report language (e.g., English); (iii) the hospital/institute which provided the report’s dataset (e.g., “default_hospital” identifies the institute which provided the datasets of reports pre-loaded in MedTAG in test mode) and (iv) the annotation mode (i.e., manual or automatic) used for the annotation process. In addition, the menu button (left-side) and the user section (right-side) are included in the header as well. It is worth noting that when the automatic annotation mode is active the users visualize the automatic annotations generated by the built-in annotation module. Any user edit concerning the automatic annotations is also replicated in the user profile, available for further edits in manual annotation mode. The user section shows the current username along with the *Logout* button. Below the header, the interface body is divided into two sections: the diagnostic report and the annotation section. The first one (left-side) shows the information regarding the textual document, that in the case of a medical report may contain the diagnosis and the patient’s information. Users can navigate between documents using either the keyboard arrows or the *next* and *previous* buttons. The annotation section (right side) shows the information concerning annotation labels, ontological concepts and the mentions identified in the selected document.

**Fig. 7:**
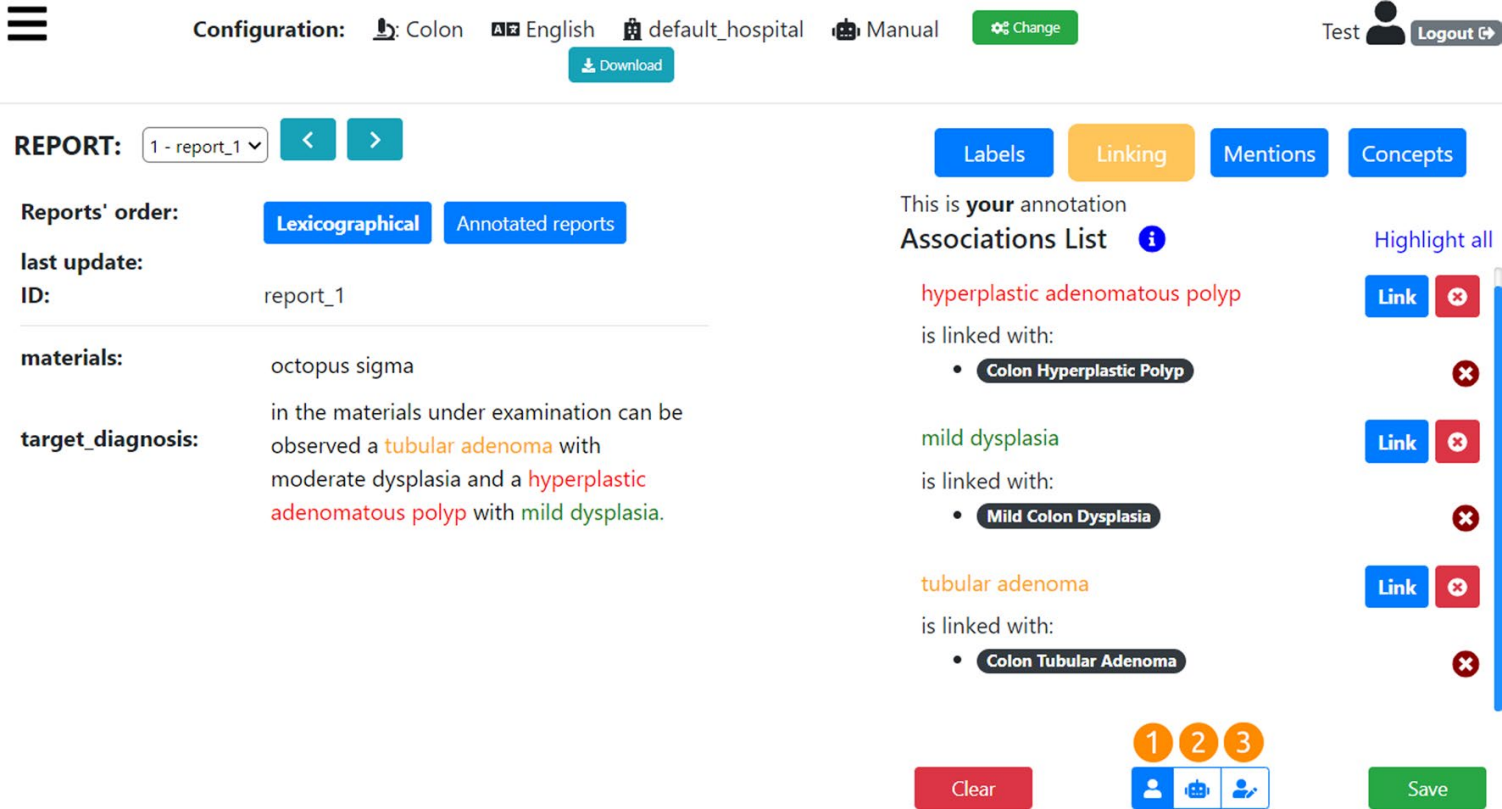
MedTAG main interface in test mode with default configuration: clinical case set to “Colon cancer”, reports’ language set to English, reports’ institute/hospital set to “default_hospital” (the real name has been anonymized) and the annotation mode set to manual. The annotation type active is the *Linking* one. Three mentions have been identified and linked to the corresponding concepts: (i) *hyperplastic adenomatous polyp* is linked to *Colon Hyperplastic Polyp*; (ii) *mild dysplasia* is linked to *Mild Colon Dysplasia*; and (iii) *tubular adenoma* is linked to *Colon Tubular Adenoma*

MedTag allows the users to use four different annotation types that can be activated alternatively by clicking on the corresponding buttons: (i) *Labels* is a form of document-level annotation where the reports are classified into predefined categories, (ii) *Mentions* where the user selects words in the text of the reports, (iii) *Linking* where the identified mentions are linked to ontological concepts, and (iv) *Concepts*, another form of document-level annotation, where the reports are annotated with ontological concepts not strictly tied to specific mentions.

In Fig. 6 the *Labels* action is activated. We can notice three selected labels: “Cancer”, “Adenomatous polyp - low grade dysplasia” and “Hyperplastic polyp”. The labels describe properties or attributes that apply to the whole document, such as the presence or the absence of cancer in the diagnosis of a clinical report. The set of labels used for the document-level annotation process, are provided by the user during the configuration phase, as previously discussed.

In Fig. 7 the *Linking* action is activated. We can see three selected multi-word mentions in the text: “tubular adenoma”, “hyperplastic adenomatous polyp’ and “mild dysplasia”. These mentions are linked to concepts taken from an histopathological ontology: (i) *hyperplastic adenomatous polyp* is linked to *Colon Hyperplastic Polyp*; (ii) *mild dysplasia* is linked to *Mild Colon Dysplasia*; and (iii) *tubular adenoma* is linked to *Colon Tubular Adenoma*.

The ontological concepts linked to the mentions can be selected via a drop-down menu (that, in turn, can be divided into semantic areas) or manually typed in a text field; in the latter case, the user is aided by auto-completion facilities.

To add a new mention, a user can click on any text token. After clicking on a text token, it gets highlighted with a new color, and the neighbor tokens turn highlighted as well, meaning that they could be selected as part of the current mention. All the mentions are highlighted with a different color in the document text and in the list of mentions for fast detection. The users can add, edit and delete the associations at will. Moreover, every time an action is performed, all the concerning information is asynchronously saved in the database; there is also manual saving via the *Save* button. Users can delete (after confirmation) all the annotations related to the current action button selected by clicking on the *Clear* button.

MedTAG enables the team members to collaborate during the annotation process. In particular, users can see anytime the annotations done by other team members for each clinical report by clicking on the button (3) of Fig. 6. This feature is handy in case of annotation uncertainty (e.g., which concepts to associate to an identified mention). To attain high-quality annotations, users can take advantage of the expertise and work other team members have previously done. In addition, users can visualize the automatic annotations made by the robot user - i.e., the automatic annotation module SKET - by clicking on button (2) of Fig. 6. Moreover, the users can consult and edit the automatic annotations so that new edits are automatically copied in the user profiles for further modifications in manual annotation mode. Hence, users can take advantage of automated annotation facilities to reduce the annotation workload. Moreover, the admin can oversee the overall annotation process from the *Team members’ statistics* section of the control panel. This section provides the admin user an overview of the annotation work carried out by each team member, providing information such as the number and the percentage of annotated reports for each use-case and annotation type. Hence, the admin can make decisions to coordinate the work of team members and keep track of the advancements in the annotation process.

Finally, users can download their annotations in different formats (i.e., BioC/JSON, BioC/XML, CSV and JSON), by clicking on the *Download* button.

**Fig. 8:**
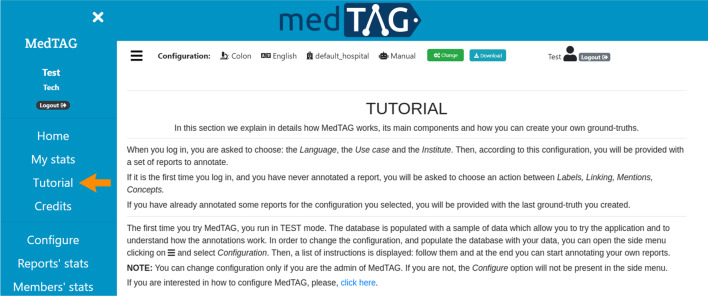
MedTAG tutorial interface. To reach the tutorial section, users can click on the *Tutorial* link in the sidebar, indicated by the orange arrow

Overall, a detailed graphical tutorial is always available to the users to learn how to use MedTAG; the *Tutorial* link is provided in the sidebar, as shown in Fig. 8.

**Fig. 9:**
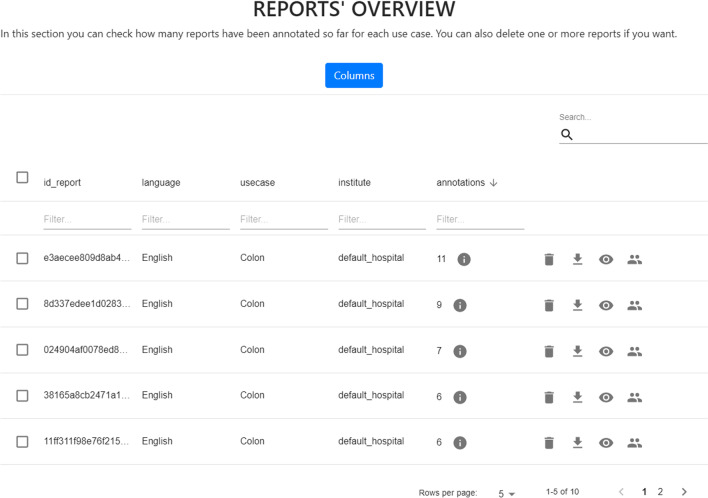
MedTAG control panel concerning the reports’ statistics. The reports are organized in an interactive table enabling the admin user to: (i) access report data; (ii) delete one or more reports; (iii) download report data including manual and automatic annotations and (iv) access the information concerning IAA and manage the majority vote procedure

**Fig. 10:**
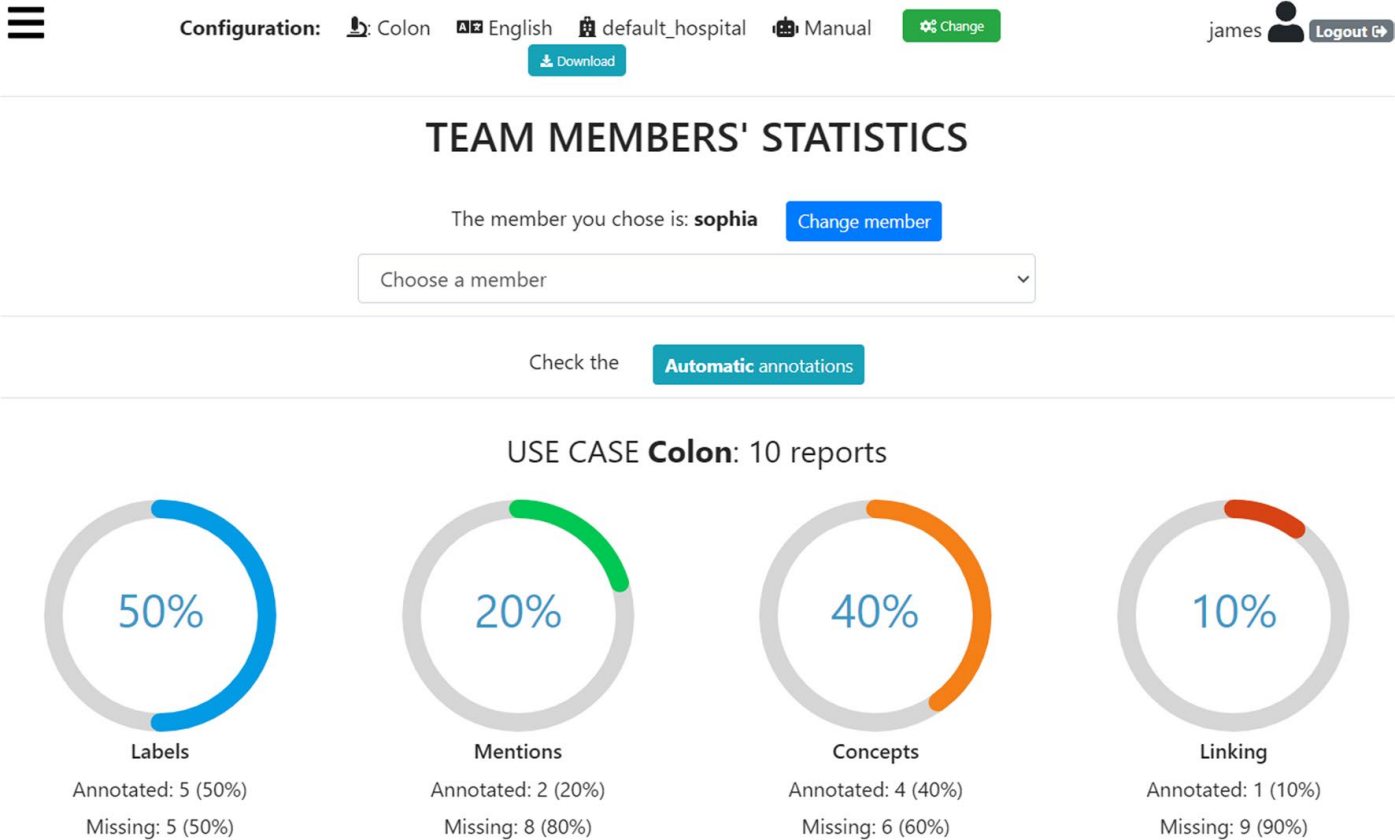
MedTAG control panel concerning the team members’ statistics. The ring charts report the annotation work carried out by each team member, so that the admin can keep track of the advancements regarding the whole annotation process

**Fig. 11:**
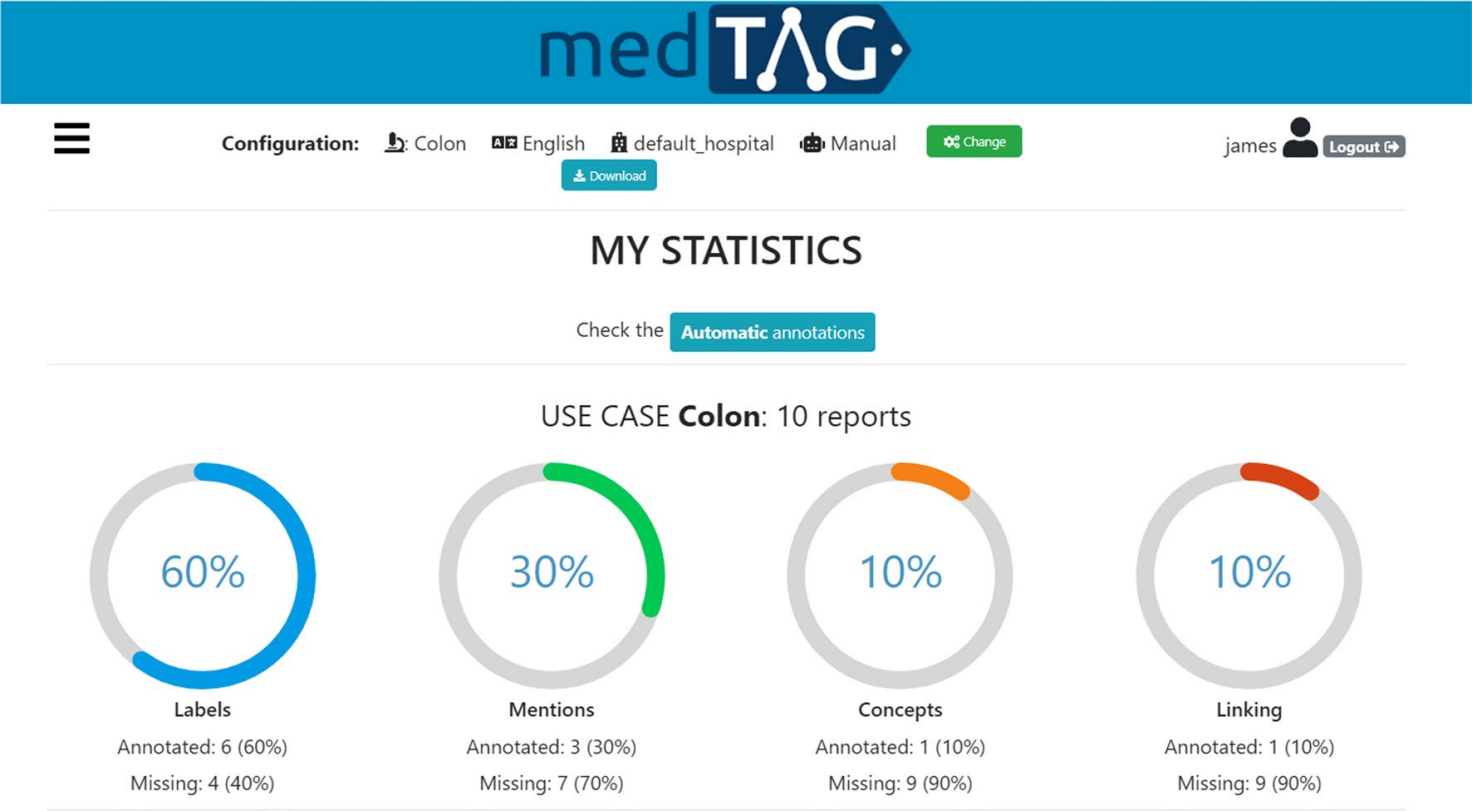
MedTAG *My Statistics* panel, providing information about the user annotation work in terms of documents annotated for each use-case

### MedTAG control panel for statistics and Inter-Annotator Agreement (IAA)

**Fig. 12:**
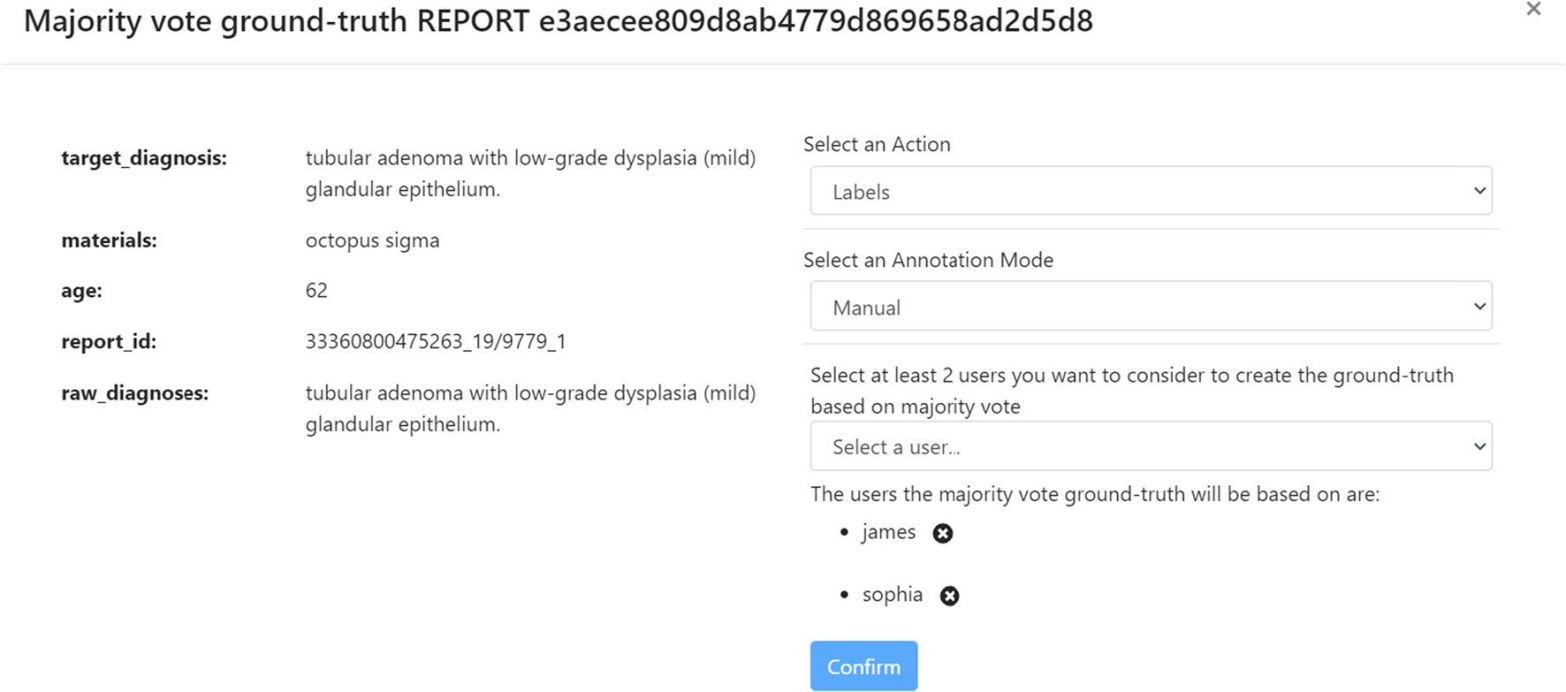
MedTAG majority vote interface. The admin can overview the selected report and choose the options of interest for the majority vote procedure, including: (i) the annotation mode; (ii) the annotation type and (iii) the team members (annotators) to consider

**Fig. 13:**
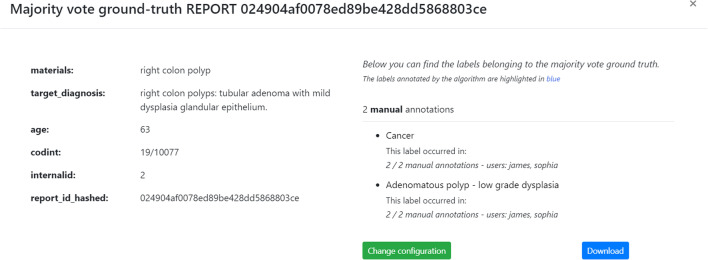
MedTAG majority vote output for the *Labels* annotation type. The admin can visualize the annotations resulting from the majority vote procedure, together with the corresponding authors. In addition, the admin can download the annotations or change the current majority vote configuration

MedTAG provides a unified interface that allows the admin user to access the annotation statistics (e.g., the number of users that annotated each report) and access the information concerning IAA for each report. It is worth noting that only the admin user can consult the statistics concerning the overall annotation process. Instead, other members can only access their statistics in the dedicated menu section *My statistics*. Figure 9 shows the control panel information organized in a dynamic table, where the admin can search, access, and filter the reports according to a selection of columns filters. Moreover, the admin can choose anytime which columns to show by clicking on the *Columns* button. The last column provides the following action buttons:

- *Delete*: enables the admin user to remove the corresponding reports.
- *Download*: allows the admin user to download either the original annotations or the ones resulting from the majority vote procedure. Moreover, the admin user can also download the automatic annotations generated by the built-in prediction system. Several download options are provided, including the output file format, the annotation mode (i.e., manual or automatic) and type (i.e., *Labels*, *Concepts*, *Mentions* and *Linking*).
- *Inspect statistics*: allows the admin user to consult the report information as well as the statistics concerning the annotations of the selected report. The annotation statistics regards all the annotation types provided in MedTAG (i.e., *Labels*, *Concepts*, *Mentions* and *Linking*) and include the number of users that identified each label, mention or concept in the report. In addition to user annotations, the interface shows the automatic annotations highlighted in blue produced by the built-in prediction system.
- *IAA and majority vote*: allows the admin user to access the information concerning IAA for each report. Figure 12 shows the pop-up modal by which the admin can specify the options for the majority vote procedure. The admin can choose from a drop-down menu which team members (annotators) to consider, as well as the annotation mode and type. The procedure returns only the annotations that achieved more than fifty percent of agreement among the annotators considered. Then, the admin can download the annotations resulting from the majority vote procedure, as shown in Fig. 13.

Figure 10 shows the *Team members’ statistics* section of the control panel, which provides the information about the advancements in the annotation work for each team member. Access to this section is restricted to the admin user. The admin can overview the annotation work carried out for each use-case and annotation type using ring charts providing information about the number of annotated reports and the corresponding percentage out of the total. Moreover, Fig. 11 shows that team members can keep track of their work by consulting the section *My statistics*, where other ring charts visually summarize the personal annotation statistics.

## Results and Discussion

MedTAG has been used to annotate diagnostic reports to produce both training and test annotated data. In particular, a specific instance of MedTAG (ExaTAG) has been used to generate more than seven thousand annotated reports and more than eight thousand annotations overall. ExaTAG^10^ is an instance of MedTAG tailored for the histopathology domain. By connecting to ExaTAG, users can try MedTAG functionalities with real (anonymized) clinical reports from the digital pathology domain without downloading and installing it. ExaTAG has been customized to meet the needs of the physicians and experts concerning three cancer use-cases (i.e., cervix, colon, and lung cancer) within the ExaMode H2020 EU project.^11^ Physicians and experts have used ExaTAG to annotate the diagnostic reports from two healthcare institutions, namely, the *Azienda Ospedaliera Cannizzaro, Catania (Italy)* and the *Radboud University Medical Center, Nijmegen, (The Netherlands)*. For the time being, ten annotators between physicians and experts have annotated thousands of medical reports in three languages (Dutch, English, and Italian). Table 1 reports some statistics about the manual annotation process conducted so far. Instead, Table 2 shows the number of automatic annotations done by SKET (i.e. the automatic annotation module) for each annotation type and use-case.

**Table 1:** Number of diagnostic reports annotated per language and use-case

Language	Use-case			
	Cervix cancer	Colon cancer	Lung cancer	Total
Dutch	–	889	–	889
English	2361	–	–	2361
Italian	1828	239	2005	4072
Total	4189	1128	2005	7322

**Table 2:** Number of labels, concepts, mentions and links (mention - concept) automatically annotated per use-case

Annotation type	Use-case			
	Cervix cancer	Colon cancer	Lung cancer	Total
Labels	16,033	9309	2066	27,408
Concepts	12,936	11,932	2336	27,204
Mentions	12,070	10,926	2336	25,332
Linking	12,936	11,932	2336	27,204
Total	53,975	44,099	9074	107,148

### Biomedical annotation tools comparison

The biomedical annotation tools selected for the comparison, according to the five requirements presented above, are BioQRator [25], ezTag [26], MyMiner [27], tagtog [28] and TeamTat [35]. Moreover, we also consider brat [39] and INCEpTION [36] because they are used by the biomedical community in some settings. Figure 1 shows that several of the considered tools lack T6 (license allowance to modify and redistribute the tool) and F1 (support for overlapping mentions). Almost half of the tools (three out of seven) lacks T2 (availability of the source code), F2 (support for document-level annotation), F11 (support for IAA), F12 (data privacy) and F13 (multilingual support). In contrast, MedTAG satisfies: (T6) MedTAG is provided through the MIT license, permitting the use, modification and distribution of the tool free of charge; (T2) the source code of MedTAG is publicly available^12^; (F12) MedTAG enables the utilization of data on a local system without any sharing with external servers, thus ensuring data privacy; (F2) MedTAG supports two types of document-level annotations, namely, label and concept annotations. The label annotation feature allows the user to tag a document according to a customizable set of labels.

The concept annotation feature allows the users to mark a document as pertinent for one or more ontological concepts. Users can leverage the auto-complete feature to search for the relevant concepts to assign. Note that, as analyzed in [34], only a tiny minority of annotation tools on the market fully support document-level annotation. For instance, MyMiner supports document annotation, but due to limits in the customization process, the annotators must re-define the labels every time new documents are added to the system. Moreover, most of the other annotation tools allow the users to provide document-level annotations only using some workaround such as zero-width annotations and annotations of pre-defined placeholders placed at the beginning or at the end of the document to annotate. However, this practice is additional overhead that further complicates and slows down the annotation process; (F10) MedTAG supports users and roles.

MedTAG is distributed as a Docker container, thus it can seamlessly be deployed in a local environment or a remote cloud solution. Therefore, the administrator can choose whether to grant MedTAG access to annotators only within a local network or “worldwide”; (F13) MedTAG provides multilingual support. It allows the users to annotate the same document (same document identifier) in different languages.

When dealing with thousands of biomedical documents to annotate, time is crucial. Hence, web-based annotation tools provided with the modality of *Software as a service* (SaaS) are not necessarily the best solution in this context due to possible network delays. For instance, network delays might be experienced when uploading high volumes of data. A local installation can avoid network delays and operate better in the case of large corpora to be annotated. However, several annotation tools present difficulties about the installation process, such as lack of documentation or dependency issues, as stated in [34]. For instance, tagtog can be installed locally only in its commercial version, whereas ezTAG and TeamTat can be installed free of charge. Still, the procedure could be quite complex for the not technology-savvy; ezTag and TeamTat require the user to install and configure some frameworks and software packages manually (e.g., Ruby, Rails, and MySQL) as prerequisites. In contrast, MedTAG provides an easy installation procedure; it requires the user only to execute the docker-compose up command (provided that Docker is installed). The MedTAG installation procedure is available and thoroughly described online.^13^

Note that TeamTat provides high-level inter-annotator agreement statistics since the project manager can calculate the agreement among annotators. In contrast, MedTAG provides fine-grained statistics by allowing the users to access the information concerning IAA for each report and to download the annotations resulting from the majority vote procedure. For this reason, we consider the criterion (F11) partially satisfied by TeamTat (see Fig. 1). TeamTat supports the annotation of documents compliant with the Unicode Standard, meaning that documents with special characters are visualized and annotated correctly. However, TeamTat does not provide additional facilities to manage, organize and search documents according to their languages (unless using a specific workaround such as creating language-specific document collections). In contrast, MedTAG allows the users to organize and filter documents according to their languages out-of-the-box; no additional configuration or effort is required. For this reason, we consider (F13) partially satisfied by TeamTat and entirely by MedTAG.

Several biomedical tools let the users upload biomedical documents by using tool-specific procedures and formats. For instance, BioQRator and ezTag only accept medical documents in BioC format. Despite BioC being a well-established file format in the biomedical domain, adopting it as the only valid format poses hindrances to annotating biomedical documents in other formats. For instance, narrative clinical reports are usually available in an unstructured format, such as plain text. Thus, to use them in BioQRator and ezTag, they need to be converted in BioC format in advance. In contrast, MedTAG allows the users to provide the medical documents as customizable CSV files, letting the user decide and set up which fields to display and annotate. This feature turns out to be helpful, especially when dealing with high volumes of long biomedical documents, where changing data format is not always a feasible or reasonable operation for annotators.

For what concerns the general-purpose annotation tools - i.e., brat and INCEpTION - they are substantially different from MedTAG. For instance, brat [39] is a well-established web-based annotation tool specifically suited for entity and relationship annotations. It has been extensively used for the annotation of biomedical projects [41–45]. Brat is not available for online use; it requires to be installed locally in a UNIX-like environment. Hence, the procedure could be complex for not technology-savvy users, as stated in [34]. In contrast, MedTAG is provided as a portable and easy-to-run Docker container. Users can configure brat via plain-text schema configuration. Moreover, users can import raw documents and export the annotations in plain-text format. Conversely, MedTAG provides support for several file formats such as BioC/JSON and BioC/XML, which are standard formats for the annotations in the biomedical domain. In addition, MedTAG also provides several other features that brat currently does not support, as (T3) online availability; (F2) support for document-level annotation; and, (F6) integration with PubMed.

INCEpTION is another general-purpose tool used also by the biomedical community [46–49]. It is an open-source web-based annotation tool both available online and for local installation. For the local use, it requires Java, as described in the online documentation.^14^ Figure 1 shows that INCEpTION covers most of the considered criteria (21 over 22). For instance, it provides active learning facilities to improve suggestions over time in a human-in-the-loop environment and a comprehensive set of features to adapt to different annotation scenarios. However, the INCEpTION interface provides several functionalities not specifically designed for the biomedical domain, which can be perceived as redundant by the biomedical community. Moreover, to achieve annotation flexibility, INCEpTION introduces additional levels of abstraction that increase the complexity of the annotation task, thus resulting potentially not within reach of not technologically-savvy users. For instance, document-level annotation is, at the time of writing, an experimental feature that needs to be explicitly enabled by manually editing a settings file. Moreover, to enable document-level annotations, the user must define a “Document metadata” annotation layer in the project settings. For such a reason, we judge the criterion (F2) as partially satisfied by INCEpTION (see Fig. 1). In contrast, MedTAG provides document-level annotation facilities off-the-shelf since no additional configuration is required. In addition, MedTAG provides native PubMed integration facilities - i.e., users can annotate PubMed titles and abstracts – whereas INCEpTION employs a third-party tool (i.e., PubAnnotation [50]) to retrieve the documents to annotate from PubMed Central, as stated in [51].

### Quantitative comparison of biomedical annotation tools

To quantitatively assess MedTAG performance, we conducted several experiments designed to evaluate MedTAG concerning two annotation tasks: document-level annotation and mention identification. The first one concerns annotations that refer to the whole document, such as labels describing the overall document content (e.g., the “cancer” label may indicate whether a clinical report suggests a cancer condition). Instead, mention identification regards entity mentions identified in the textual content of a document. The annotation tools are compared regarding the number of actions and elapsed time required to complete the overall annotation process. To the best of our knowledge, this is the first available quantitative evaluation of biomedical annotation tools. The analysis we conducted considers a set of web-based biomedical annotation tools - i.e., ezTag, MedTAG, MyMiner, tagtog and TeamTat - evaluated on a sample of one hundred documents, randomly chosen from a real dataset concerning the digital pathology domain (i.e., clinical reports related to colon cancer). For the comparison, we consider only web-based publicly available tools since many biomedical annotation tools are not available for local installation or are not easy to install for not technologically savvy end-users. It is worth noting that our analysis does not focus on usability and *Human-Computer Interaction* (HCI) aspects (e.g., *User Experience*(UX) and interface look and feel) that may vary subjectively. Nevertheless, the latter are essential points that should be treated with specific user studies. In contrast, we focused on the annotation work regarding the number of actions (e.g., mouse clicks and keys pressed) and elapsed time to achieve the same annotations in different tools. To perform a fair comparison, we used automatic agents (web robots) designed to annotate using the same annotation speed - i.e., exact time to simulate a mouse click or a key pressed for each annotation tool. The automatic agents have been implemented using the Python Web automation library Selenium.^15^ The source code of the automated agents used for the experiments is publicly available.^16^ Since the automatic agents are generally way faster than any human annotator, we introduced a short delay (about two hundred milliseconds) between two consecutive actions, which is also necessary to avoid overloading the server with too many requests.

Tables 3 and 4 show the experimental results in terms of the number of actions and elapsed time for annotating one hundred documents. The elapsed time for each tool was recorded forty times; the resulting mean value and standard deviation are reported in the tables. Table 3 shows the performance analysis concerning the document-level annotation task. For the latter task, we considered three tools - i.e., MedTAG, MyMiner, and tagtog - since ezTag does not support document-level annotation, whereas TeamTat provides different document-level annotation facilities. In particular, TeamTat allows us to annotate entities in different documents and then to create relationships between them; this is different from the functional criterion (F2), indicating whether the users can specify labels at the document-level. For this reason, we consider the latter criterion only partially satisfied. The experiments concerning document-level annotation consist of assigning one label for each document to annotate. The labels, mentions, and documents used for testing are publicly available^17^ for reproducibility purposes. Table 3 shows that MyMiner requires fewer actions than other tools to achieve the same annotations, whereas MedTAG turns out to be the fastest tool in terms of elapsed time. Nevertheless, the time difference between MyMiner and MedTAG is about ten seconds, which is negligible considering different server response times. According to Table 3, tagtog requires more actions and time than other tools to complete the annotation process. However, these results are motivated considering that tagtog is one of the most flexible annotation tools and allows to specify whether a document label is *true*, *false* or *unknown*. To this aim, tagtog allows users to choose the correct value from a drop-down menu for a document label. Thus, the users have to click on the drop-down menu two times: the first one to open the pop-up menu and the second for the value selection. In contrast, MyMiner and MedTAG require just one click on a checkbox, based on the assumption that a label may apply for a document or not (the *unknown* state is not allowed). Moreover, MyMiner requires fewer actions than MedTAG to complete the annotation process since it automatically moves on to the following document to annotate after the user selection. However, MyMiner does not allow to specify more labels for a document. In contrast, MedTAG goes beyond this limitation and allows to specify of several labels at the same time for each document. Thus, users can decide on their own when to move on to the next document to annotate.

Table 4 shows the performance analysis concerning the mention identification task. The experiments concerning mention identification consist of identifying entity mentions within the documents’ textual content. To this aim, we used a set of pre-identified mentions for each of the documents considered. According to Table 4, the tools with the lowest number of actions required are ezTag and TeamTat, whereas MyMiner and MedTAG are the fastest tools in terms of elapsed time. TeamTat and ezTag achieved comparable performance since they are similar in terms of functionalities provided. The experimental results show that MyMiner is the fastest tool in terms of elapsed time. MyMiner provides a neat interface that requires low network resources and bandwidth to work, thus reducing loading time and making the annotation process faster. However, it lacks several functionalities such as (i) support for users and teams, (ii) availability for local installation, and (iii) data privacy (upload of the documents to annotate is required) that could be relevant for the needs of the biomedical community. In contrast, MedTAG is designed to be portable (i.e., local installation is available) and flexible; it provides annotation facilities, such as schema configuration, that allow users to customize the annotation experience. Moreover, MedTAG is faster than other tools, even if it requires more actions. A possible explanation could be the different mention annotation functionality. Indeed, most of the annotation tools allow identifying entity mentions within the text using drag-and-drop facilities. In contrast, MedTAG enables users to annotate mentions with a single click on each text token. The latter facility turns out to be convenient in short mentions, whereas drag-and-drop is more suitable in the case of long ones.

To summarize, we quantitatively compared a set of web-based biomedical annotation tools on two tasks: document-level annotation (one label per document) and mention identification. We conducted several experiments to assess each annotation tool regarding the number of actions and elapsed time required to complete the overall annotation process. From the experimental results emerge that, depending on the task, some tools perform better than others. Despite the higher number of actions required to complete the annotation process, MedTAG turns out to be faster than other tools, especially for the document-level annotation task.

Finally, it is worth noting that the present study focuses on evaluating a set of biomedical annotation tools only on physical aspects such as the number of actions and elapsed time required to annotate all the documents considered. Hence, we do not consider several critical human-centric factors (e.g., UX and HCI) that should be investigated in dedicated usability studies.

**Table 3:** Document-level annotation performance analysis in terms of number of actions (e.g. mouse clicks and keys pressed) and elapsed time required to complete the whole annotation process

Tool	Number of actions	Elapsed time in seconds (mean)	Standard deviation in seconds
MedTAG	200	46.840	0.803
MyMiner	100	56.677	0.416
tagtog	400	205.740	5.471

**Table 4:** Mention-level annotation performance analysis in terms of number of actions (e.g. mouse clicks and keys pressed) and elapsed time required to complete the whole annotation process

Tool	Number of actions	Elapsed time in seconds (mean)	Standard deviation in seconds
MedTAG	519	159.337	0.479
ezTag	307	260.340	0.576
MyMiner	414	114.390	1.507
tagtog	404	304.692	10.067
TeamTat	307	271.577	1.542

## Conclusions

We presented MedTAG, a customizable, portable, collaborative, web-based biomedical annotation tool. We described an instance of MedTAG adopted in the histopathology domain, where MedTAG has been used by physicians to annotate more than seven thousand clinical reports in three languages (Dutch, English and Italian), from two health-care institutions. MedTAG is provided as a docker container to make it distributable, platform-independent and easy to install/deploy. We designed MedTAG according to the five requirements (i.e. available, distributable, installable, workable and schematic) defined in a recent extensive review of manual annotation tool [34]. Moreover, MedTAG satisfies 20 over 22 criteria defined in the same study.

The key points of strength of MedTAG are: (i) fast and easy installation because only one command is necessary to install it in less than 10 min on a current notebook; (ii) cross-platform support since MedTAG can be installed in every platform supporting docker; (iii) a collaborative web-based platform supporting users and roles; (iv) broad data formats support including BioC/JSON, BioC/XML, CSV, and JSON; (v) support for schema configuration where the users provide the documents to annotate by using custom CSV files and can decide which fields to display and annotate.

### Limitations and future work

MedTAG, as the name suggests, is a customizable annotation tool for the biomedical domain; Thus, it is not intended for general-purpose annotations since the users could not exploit domain-specific features such as automatic annotation. It is worth noting that the automatic annotation is currently provided for three cancer use-cases (i.e., cervix, colon, and lung cancer). Nevertheless, we plan to extend the automatic annotation support for other use-cases according to the needs of the biomedical community. The integration of SKET as an automated annotation tool shows the flexibility of MedTAG and how annotation automation may work with MedTAG. Another limitation concerns the file format of the input documents since MedTAG currently supports only plain-text documents. We believe that PDF annotation would be particularly useful, especially when dealing with scientific paper annotation. Hence, we plan to include this feature in the future version of MedTAG. For the time being, MedTAG does not support both overlapping mentions (also known as multi-label annotations) and relationship annotations that are left as future work. Indeed, even if MedTAG allows assigning multiple concept labels to the same mention, it is currently impossible to annotate any sub-mention. Finally, it is worth noting that even if MedTAG is designed for the biomedical domain, it could also be used for general-purpose annotation as long as a suitable schema configuration is provided. As future work, we plan to enrich MedTAG by adding (i) the support for overlapping mentions; (ii) the support for relationship annotations; (iii) the support for active learning capabilities; (iv) the support for PDF annotation; (v) the automatic annotation support for other use-cases relevant for the biomedical community. Thereby, we aim to improve MedTAG according to the biomedical community’s needs and foster further developments in this field.

## Availability and requirements

- Project name: MedTAG
- Project home page: https://github.com/MedTAG/medtag-core
- Operating system(s): Platform independent.
- Other requirements: Docker and docker-compose
- License: MIT License
- Any restrictions to use by non-academics: No

## Data Availability

The full source code of MedTAG is available at https://github.com/MedTAG/medtag-core. An instance of MedTAG (i.e. ExaTAG) is publicly available for testing it, using the following credentials: username and password *Test*.

## Abbreviations

EHRs: Electronic health records
HCI: Human-computer interaction
IAA: Inter-annotator agreement
IE: Information extraction
ML: Machine learning
NCIT: National cancer institute thesaurus
NER+L: Named entity recognition and linking
NLP: Natural language processing
PMC: PubMed central
SaaS: Software as a service
SKET: Semantic knowledge extractor tool
UI: User interface
UX: User experience

## Footnotes

1 We report details about the main features considered in [34] to ease the comprehension of our analysis.

2 https://github.com/ExaNLP/sket/.

3 http://examode.dei.unipd.it/ontology/.

4 https://reactjs.org/.

5 https://www.djangoproject.com/.

6 https://docs.docker.com/engine/reference/commandline/docker/.

7 https://docs.docker.com/compose/.

8 https://github.com/MedTAG/medtag-core/tree/main/MedTAG_Dockerized.

9 https://github.com/MedTAG/medtag-core.

10 http://w3id.org/exatag/ access granted with username and password *Test* for reviewing purposes.

11 https://www.examode.eu/.

12 https://github.com/MedTAG/medtag-core/.

13 https://github.com/MedTAG/medtag-core/tree/main#installation.

14 https://inception-project.github.io/documentation/.

15 https://www.selenium.dev/.

16 https://github.com/MedTAG/medtag-core/tree/main/benchmark.

17 https://github.com/MedTAG/medtag-core/tree/main/benchmark/datasets.
